# Clinical feasibility study of transcatheter edge-to-edge mitral valve repair in dogs with the canine V-Clamp device

**DOI:** 10.3389/fvets.2024.1448828

**Published:** 2024-12-09

**Authors:** Brianna M. Potter, E. Christopher Orton, Brian A. Scansen, Katie M. Abbott-Johnson, Lance C. Visser, I-Jung B. Chi, Evan S. Ross, Bruna Del Nero, Lalida Tantisuwat, Ellen T. Krause, Marlis L. Rezende, Khursheed Mama

**Affiliations:** Department of Clinical Sciences and James L. Voss Veterinary Teaching Hospital, College of Veterinary Medicine and Biomedical Sciences, Colorado State University, Fort Collins, CO, United States

**Keywords:** mitral regurgitation, degenerative mitral valve disease, myxomatous mitral valve disease, transapical intervention, transcatheter mitral valve repair

## Abstract

**Objective:**

To determine procedural feasibility, safety, and short-term efficacy in dogs with severe degenerative mitral regurgitation (MR) undergoing transcatheter edge-to-edge repair (TEER) with a canine-specific device.

**Design:**

Prospective, single-arm (uncontrolled), single-institution clinical feasibility study.

**Animals:**

Fifty client-owned dogs with severe degenerative MR operated over a 28-month period.

**Methods:**

TEER was performed using the canine mitral V-Clamp via a transapical approach using transesophageal echocardiographic and fluoroscopic guidance. Indices of MR severity were determined by echocardiography the day before and 2 to 3 days after the procedure.

**Results:**

Procedural feasibility was 96% based on delivery of at least one device in 48 of 50 dogs. There were no procedural deaths. Procedural safety was 96% based on survival to hospital discharge in 48 of 50 dogs. Euthanasia in 2 dogs prior to hospital discharge was due to damage of the mitral valve and worsened MR after the procedure. Device-related adverse event rate was 6.3% based on 3 events (single-leaflet device detachment, locking failure, locking failure with device embolization) in 59 implanted devices. All three events were nonfatal and successfully treated with a second device. Median regurgitant volume (mL/kg) decreased (*p* < 0.001) from 2.3 [1.9, 3.1] to 1.1 [0.3, 1.8]. Median effective regurgitant orifice area (cm^2^/m^2^) decreased (*p* < 0.001) from 0.60 [0.40, 0.80] to 0.25 [0.10, 0.50].

**Conclusion and clinical importance:**

Initial feasibility results support continued development of TEER as a procedurally feasible, relatively low-risk, and low morbidity treatment for degenerative MR in dogs. Operator experience and case selection are likely to be important components of success of this technique. Evidence of short-term efficacy is promising but needs to be verified with longer-term follow up.

## Introduction

Therapeutic options for degenerative mitral valve disease (DMVD) in dogs are largely limited to medical management (1, 2). Surgical mitral valve repair is performed at a few centers worldwide, although availability and high expense limit this option (3–5). Additional procedural strategies are needed to address severe mitral regurgitation (MR) in this large population of dogs.

The concept of edge-to-edge mitral valve repair was first proposed by Ottavio Alfieri as a surgical option to address prolapse of the anterior mitral leaflet in humans (6, 7). The success of this surgical technique led to the development of the MitraClip device for transcatheter edge-to-edge repair (TEER) of the mitral valve in humans. The first feasibility EVEREST trial of the MitraClip device in humans (8, 9) was followed by two pivotal trials, EVEREST II (10) and COAPT (11). These studies paved the way for what has become a widely accepted and successful option for the treatment of severe MR in humans. An additional TEER device, the PASCAL device, has been found to be non-inferior to MitraClip for major adverse events among humans with degenerative MR and prohibitive surgical risk (12). The success of TEER in humans prompted the development of a canine-specific TEER device called the Canine Mitral V-Clamp. An early study demonstrated procedural feasibility of the device in eight dogs in American College of Veterinary Internal Medicine (ACVIM) stage B1 (13). Based on these results, a prospective clinical feasibility study of the V-Clamp device was undertaken in dogs with severe degenerative MR in ACVIM stages B2, C, and D.

## Materials and methods

### Study design and patient selection

**Figure 1:**
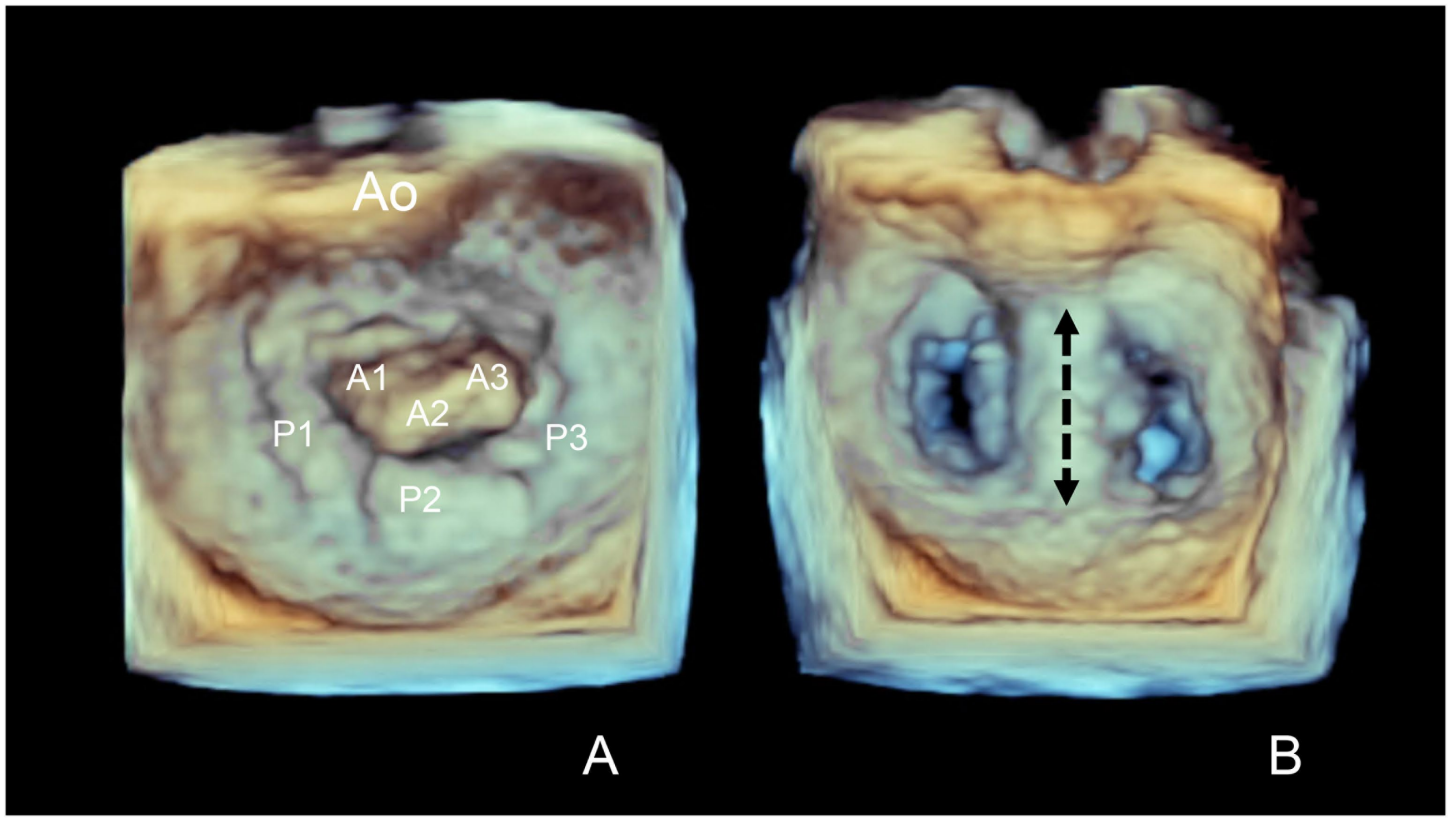
Transesophageal echocardiographic *en face* images of the mitral valve before (A) and after (B) transcatheter edge-to-edge mitral valve repair (TEER). Wide prolapse of the A2 segment of the mitral valve is present before TEER. The TEER procedure results in a double orifice mitral valve with the clamp location positioned across the A2 and P2 segments (dashed arrowed line). Ao, aorta.

A prospective, single-arm (uncontrolled), single-institution clinical feasibility study of TEER using the Canine Mitral V-Clamp^1^ device was conducted in dogs with severe degenerative MR. Study enrollment period was January 2021 through April 2023 and included all initial dogs undergoing the procedure at this institution. The inclusion criterion was severe degenerative MR based on criteria adapted from the American Society of Echocardiography integrative guidelines for evaluation of native valvular regurgitation (14). The first cases were ACVIM stage C or D (1). After initial procedural success, stage B2 cases were added as long as dogs met predefined criteria for severe MR as defined below in the echocardiography section. Predefined exclusion criteria were severe pulmonary hypertension with estimated tricuspid regurgitation pressure gradient >80 mmHg, non-cardiac disease judged likely to influence six-month survival, and evidence of active systemic infection or immune-mediated disease. Based on three available V-Clamp sizes (14 mm, 16 mm, 18 mm), the mid-systolic anterior–posterior (AP) mitral annular diameter measured from the left apical inflow-outflow view on transthoracic echocardiography (15) was between 14 mm and 22 mm. Anatomic eligibility criteria was leaflet prolapse or flail of the central (A2 and/or P2) mitral valve segments (Figure 1A).

The study endpoints were: (1) procedural feasibility defined as delivery of at least one clamp, (2) procedural safety defined as survival to hospital discharge, (3) procedural efficacy based on echocardiographic assessment of MR severity at baseline and 2 to 3 days post-procedure. The study was conducted under informed client consent with approval by the Institutional Animal Care and Use Committee and the Clinical Review Board at Colorado State University. There were no modifications to the device during the course of the study.

### Echocardiography and hemodynamics

Transthoracic echocardiographic examinations were performed by a board-certified cardiologist or cardiology resident under the supervision of a board-certified cardiologist using an ultrasound unit^2^ equipped with several phased-array transducers (5–9 MHz) that were matched to the size of the dog. A standardized imaging protocol was used for each examination. All echocardiographic measurements were performed by a single board-certified cardiologist (BMP). All dogs had a standard two-dimensional, M-mode, and Doppler transthoracic echocardiographic examination performed using recommended right parasternal, subcostal, and left parasternal imaging planes (16). In addition to standard two-dimensional imaging, a left parasternal commissural view (15) was obtained along with biplane evaluation of the mitral valve on left parasternal views and three-dimensional *en face* images of the mitral valve.

The short axis left atrium to aortic dimension ratio was measured from the right parasternal short axis view at the level of the aortic root just after aortic valve closure (17). Measurements were made at the blood-tissue interface. The long axis LA was measured from a right parasternal long axis (RPLA) four-chamber view optimized for the left atrium and maximum left atrial diameter was measured mid-chamber at end-systole with a line parallel to the mitral valve annulus. The long axis LA was indexed to the aortic dimension, which was measured from a RPLA view optimized for the aortic valve. The aortic dimension was measured during early to mid-systole, between the two visible leaflets when the aortic valve was maximally opened (18). The maximum left atrial volume at ventricular end-systole was estimated from a RPLA four-chamber view optimized for the left atrium using monoplane Simpson’s method of discs and then indexed to body weight (kg) (19). The left ventricular internal dimension in diastole was measured using the right parasternal short axis view at the level of the papillary muscles and normalized to body weight (20). The left ventricular volumes were estimated from a RPLA four-chamber view optimized for the left ventricle using monoplane Simpson’s method of discs and then indexed to body weight.

Echocardiographic variables of cardiac remodeling and MR severity included left atrial and ventricular size, color Doppler assessment of jet area, E-wave velocity, regurgitant volume (RVol), and regurgitant fraction (RF). RVol and RF were estimated using the volumetric method [RVol (mL) = Total left ventricular stroke volume (SV) – Forward SV; RF (%) = RVol/Total SV] (14). Total left ventricular SV was estimated using monoplane Simpson’s method of discs from the RPLA four-chamber view (21). The forward SV was obtained using the aortic dimension from the RPLA view as described above and the aortic velocity time integral was obtained from a subcostal view. Effective regurgitant orifice area (EROA) was calculated from the calculated RVol and MR velocity time integral [EROA (cm^2^) = RVol (mL)/MR VTI (cm)] and indexed to body surface area (14, 22). Criteria of severe MR for study entry were E-wave velocity > 1 m/s, RF > 50%, and RVol >1.0 mL/kg (14, 23). Transthoracic echocardiographic variables were obtained the day before intervention and at day two or three after intervention and analyzed by a single board-certified cardiologist (BMP). Transesophageal echocardiography (TEE) was performed prior to the procedure under general anesthesia to further assess prolapse/flail location, AP mitral annulus dimension, and facilitate procedural guidance. Intraprocedural hemodynamic and TEE data included heart rate, invasive blood pressure, and spectral Doppler estimates of mean mitral inflow pressure gradient collected just prior to and 5 min after device deployment.

### Procedure

Transcatheter edge-to-edge mitral valve repair was performed under general anesthesia via a minimal-incision (3 to 4 cm) left thoracotomy and transapical left ventricular access with dogs in right lateral recumbency. The optimal intercostal space for the transapical approach was determined based on a lateral fluoroscopic view of the cardiac silhouette and transthoracic echocardiography, typically the 7th intercostal space. Intercostal thoracotomy was made at the ventral aspect of the intercostal space close to the sternum (Supplementary Video 1). The pericardium was opened and sutured to the incision to elevate the cardiac apex. The location of the left ventricular access site was determined by external blunt compression of the heart visualized with biplane TEE of the left ventricle and mitral valve. The optimal access site was determined as perpendicular to the plane of the mitral valve on both the inflow-outflow and commissural views (24). Two pledget-reinforced mattress sutures of 4–0 polypropylene were placed around the optimal puncture site and passed through tourniquets.

**Figure 2:**
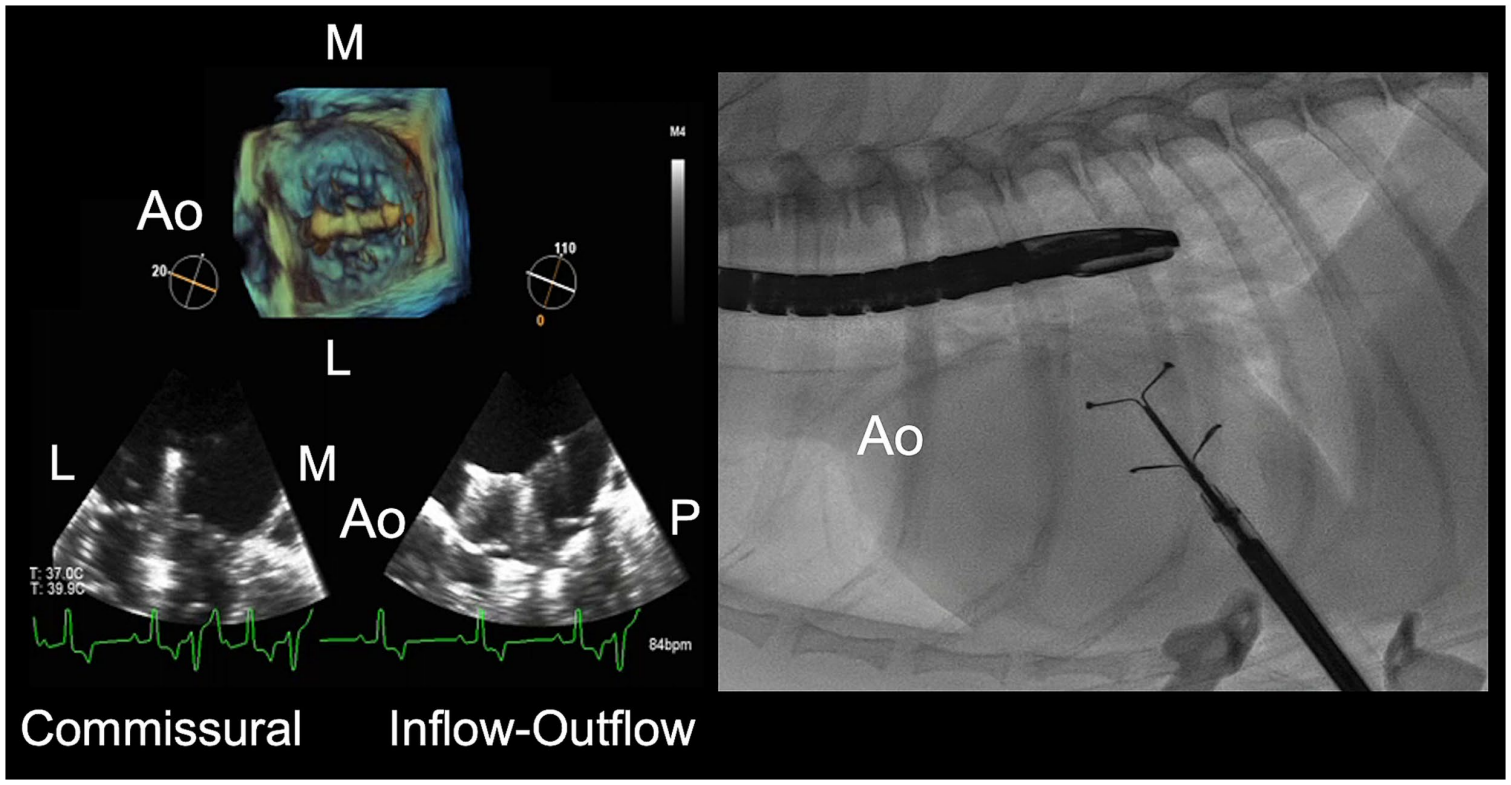
Transesophageal echocardiographic (TEE) and fluoroscopic imaging for transcatheter edge-to-edge mitral valve repair. Views for TEE include two-dimensional commissural and inflow-outflow views, and three-dimensional *en face* view of the mitral valve. The fluoroscopic view is a lateral projection of the dog in right lateral recumbency. The aorta (Ao) is oriented at 9 o’clock to provide synchronous cranial-caudal movement of the device between the TEE inflow-outflow, *en face*, and fluoroscopic views. L, lateral; M, medial; P, posterior.

The procedure was performed under fluoroscopic and TEE visualization. The typical fluoroscopic view was 10° cranial and 0° to 10° left anterior oblique on the fluoroscope (90° to 100° left ventral oblique in reference to the sternum of the patient in right lateral recumbency). Simultaneous TEE views were biplane commissural and inflow-outflow views, and a three-dimensional *en face* view of the mitral valve (Figure 2) (24). The *en face* view was oriented with the aorta at 9 o’clock to show synchronous cranial-caudal movement of the device between the *en face*, biplane inflow-outflow, and fluoroscopic views.

**Figure 3:**
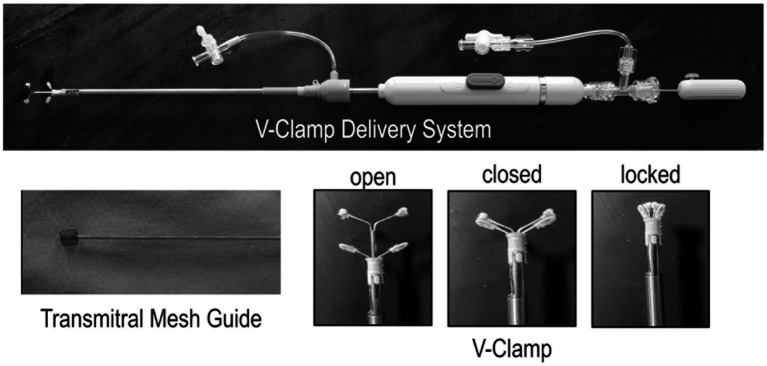
Canine Mitral V-Clamp delivery system, transmitral mesh guide, and V-Clamp. The V-Clamp has 3 positions: open, closed, and locked.

An intravenous bolus of unfractionated heparin (50 U/kg) was administered prior to accessing the left ventricle. Three device widths (14 mm, 16 mm, 18 mm) were available (Figure 3). V-Clamp device size selection was based on the mid-systolic AP mitral annulus dimension on TEE inflow-outflow view minus 0 to 2 mm (e.g., an 18 mm device for a 20 mm AP dimension). Cardiac puncture was performed with an 18 gauge over-the-needle catheter. An 0.035″ 50 cm J-tip guidewire was passed through the catheter into the left ventricle. In early cases, the J-tip wire was passed retrograde across the mitral valve under biplane TEE visualization. A 14Fr introducer and dilator was then advanced over the wire into the left ventricle and then into the left atrium. The dilator and guidewire were removed. In later cases, the 14Fr introducer was advanced over the guidewire into the left ventricle. The dilator and guidewire were removed. A specialized mitral crossing device with a soft Nitinol basket tip (Figure 3) was introduced into the 14Fr introducer and passed retrograde across the mitral valve and into the left atrium under fluoroscopic and TEE visualization. The introducer was advanced into the left atrium and the mitral crossing device was then removed (Supplementary Video 2). The V-Clamp delivery system was passed into the left atrium via the introducer. The arms of the V-Clamp were manipulated to obtain optimal medial-lateral, AP, and rotational orientations on TEE and fluoroscopic views prior to antegrade crossing of the mitral valve. The lower arms of the clamp were brought ventrally across the mitral valve to span the leaflets while maintaining optimal orientation (Supplementary Video 3). The lower arms of the clamp were then advanced dorsally toward the mitral valve to suspend the leaflets. The valve leaflets were captured by bringing the upper arms down to the valve, thereby closing the clamp. Once initial leaflet capture was confirmed based on TEE and fluoroscopy, the clamp was locked. Residual MR and mitral inflow gradient were assessed. The unclamped portion of the anterior and posterior leaflets were measured and subtracted from the pre-deployment leaflet lengths to ensure adequate leaflet capture with the aim of achieving ≥4 mm of capture for each leaflet. The leaflet lengths were measured on the inflow-outflow view in a mid-systolic frame from the hinge point of the leaflet to its insertion in the clamp via a curved distance measurement (24). The device was then released from the delivery system (Supplementary Video 4). After deployment, residual MR and mitral inflow gradient were thoroughly assessed on TEE and clamp positioning was documented on fluoroscopy. Initially only single clamp deployments were attempted. After gaining experience with single clamp deployment, a second clamp was deployed to address residual MR when MR severity remained moderate or greater as subjectively assessed on TEE with color Doppler and when there was adequate space to manipulate a second clamp lateral or medial to the first clamp. In cases where a second clamp was placed, the same basic procedure was used with deployment as close as possible to the first clamp. After deployment, the delivery system and introducer were removed, and the mattress sutures at the transapical access site were tied. A thoracostomy tube was placed and the thoracotomy site was closed.

Dogs were monitored in the hospital for 24 to 48 h. Oral clopidogrel (2 mg/kg) was initiated on the first post-operative day and continued for 3 months. Pimobendan was continued after TEER at the pre-procedural dose in all dogs. In dogs that were receiving diuretic therapy before the procedure, all dogs were continued on diuretic therapy post-procedure in the short-term. The diuretic dose was based on the clinician managing the case and the median post-operative furosemide dose at discharge was 50% [12.8, 66.6%] of the original dose.

### Statistics

Normality testing was performed with a Shapiro–Wilk test. Within-subject comparisons were performed with a paired t-test if differences of data pairs were normally distributed or a Wilcoxon rank sum test if differences of data pairs were not normally distributed. Between-subject comparisons were performed with a Student’s t-test if residuals were normally distributed or a Mann–Whitney U test if residuals were not normally distributed. Values of *p* < 0.05 were considered evidence of a difference.

## Results

**Figure 4:**
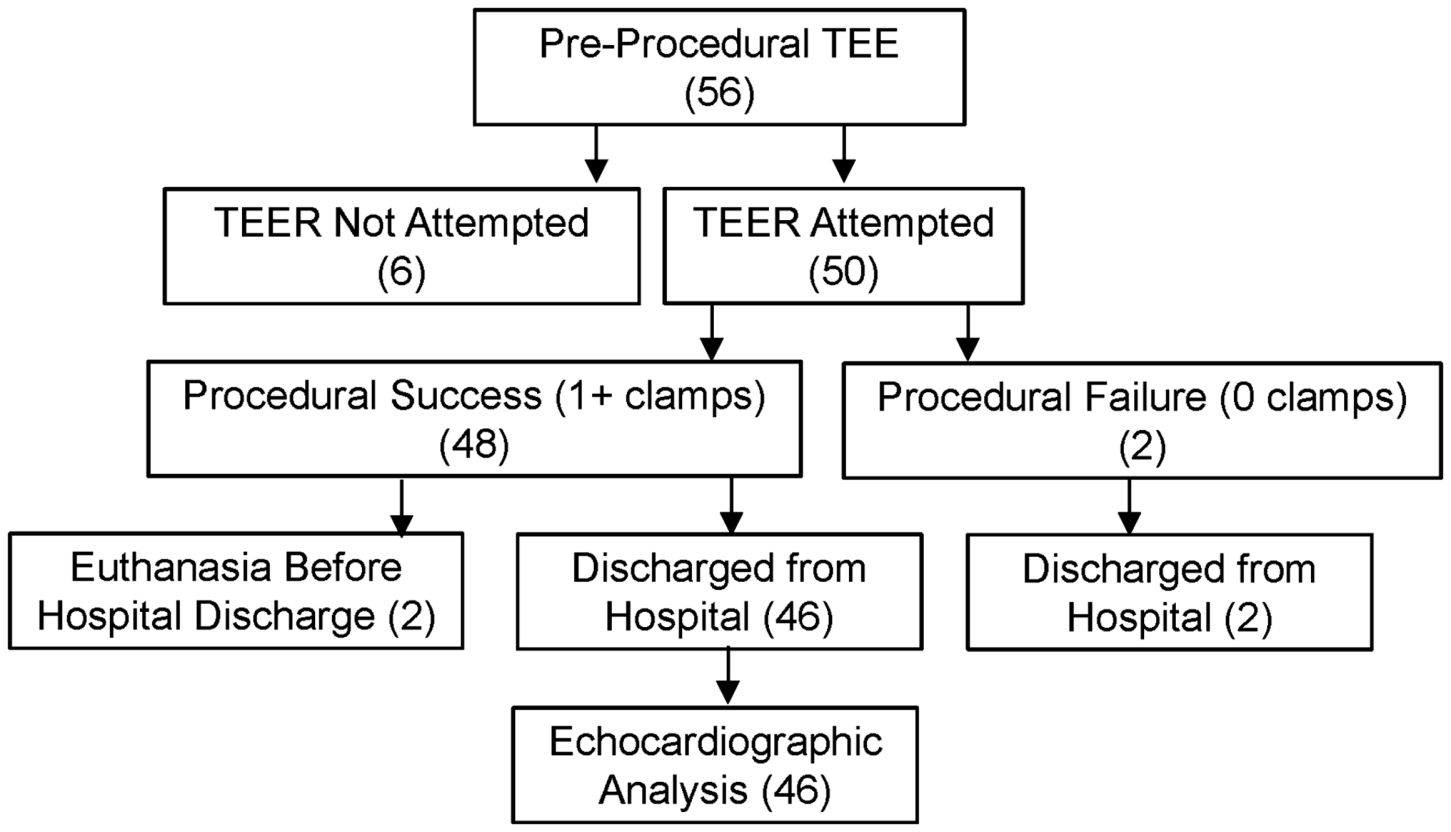
Study profile for the feasibility clinical study of transcatheter edge-to-edge mitral valve repair in dogs with the canine mitral V-Clamp device.

The study profile is shown in Figure 4. Fifty-six dogs were placed under general anesthesia for pre-procedural TEE. Six dogs were judged to be inappropriate for TEER based on unfavorable mitral valve functional anatomy (e.g., severe leaflet flail or extensive involvement of non-central segments) and/or AP dimension, and TEER was not attempted. Fifty dogs underwent the TEER procedure and are included in the analysis. Patient characteristics are reported in Table 1 and breeds included are reported in Supplementary Table A. Procedural feasibility was 96% based on successful deployment of at least one clamp in 48 of 50 dogs. The V-Clamp device was not implanted in two dogs due to failure to capture the leaflets. Both dogs were discharged from the hospital. One of these dogs died of progressive heart failure 6 weeks after the surgery. The other dog died suddenly the day after hospital discharge. Procedural safety was 96% based on survival to hospital discharge of 48 of 50 dogs. There were no intraprocedural deaths. Two dogs were euthanized before hospital discharge due to worsened MR after the procedure. Overall procedural success based on successful deployment of at least one clamp and discharge from the hospital was 92%. Median total procedure time (intubation to extubation) was 190 min [175, 205 min].

**Table 1:** Baseline characteristics of dogs undergoing transcatheter edge-to-edge mitral valve repair.

	*n* = 50
Age (years)	9.9 (8.7, 11.3)
Female (number (percent))	13 (26%)
Body weight (kg)	7.9 (6.1, 9.4)
**Breed** CKCS (number (percent))	13 (26%)
Heart rate (bpm)	118 (96, 130)
Systolic blood pressure (mmHg)	132 (120, 145)
**Cardiac rhythm** Sinus rhythm/sinus tachycardia Atrial premature complexes Ventricular premature complexes	50 (100%) 2 (4%) 1 (2%)
Acquired atrial septal defect	3 (6%)
**ACVIM Stage** B2 C < 6 months C > 6 months D	15 (30%) 25 (50%) 7 (14%) 3 (6%)
**Treatments at baseline** Pimobendan ACEi Spironolactone Diuretic Furosemide Dose (mg/kg/d) Torsemide Dose (mg/kg/d)	50 (100%) 35 (70%) 28 (56%) 35 (70%) 3.7 (3.0, 5.0) 1 (2%) 1.1
**Mitral valve characteristics on pre-procedural TEE** A2 and P2 segment prolapse/flail A1 and/or A3 segment prolapse/flail P1 and/or P3 segment prolapse/flail Mid-systolic A-P diameter (mm)	50 (100%) 27 (54%) 9 (18%) 19.5 (18.0, 21.0)

Values are reported as median (25th-centile, 75th-centile) unless otherwise stated.

ACEi, angiotensin converting enzyme inhibitor; AP, anterior posterior; CKCS, Cavalier King Charles Spaniel.

Fifty-nine V-clamps were delivered into 48 dogs. Nine dogs had two clamps delivered. One dog had three clamps delivered. Adverse device-related event rate was 6.3% based on three nonfatal events. All three events were attributed to operator error and inexperience. In two events, the clamp became unlocked shortly after deployment. In one of these incidents, the clamp remained clamped to the valve. In the other, the clamp separated, released from the valve, and embolized to the aorta. The embolized clamp was not removed. This patient remained asymptomatic post-operatively and was maintained on clopidogrel long-term and no anti-coagulants were necessary. In both cases, a second clamp was successfully deployed during the index procedure. The third event was a single-leaflet device detachment 30 h post-procedure. A second clamp was successfully deployed during a second procedure 2 days after the index procedure. Additional details for adverse events are included in Supplementary Table B.

**Figure 5:**
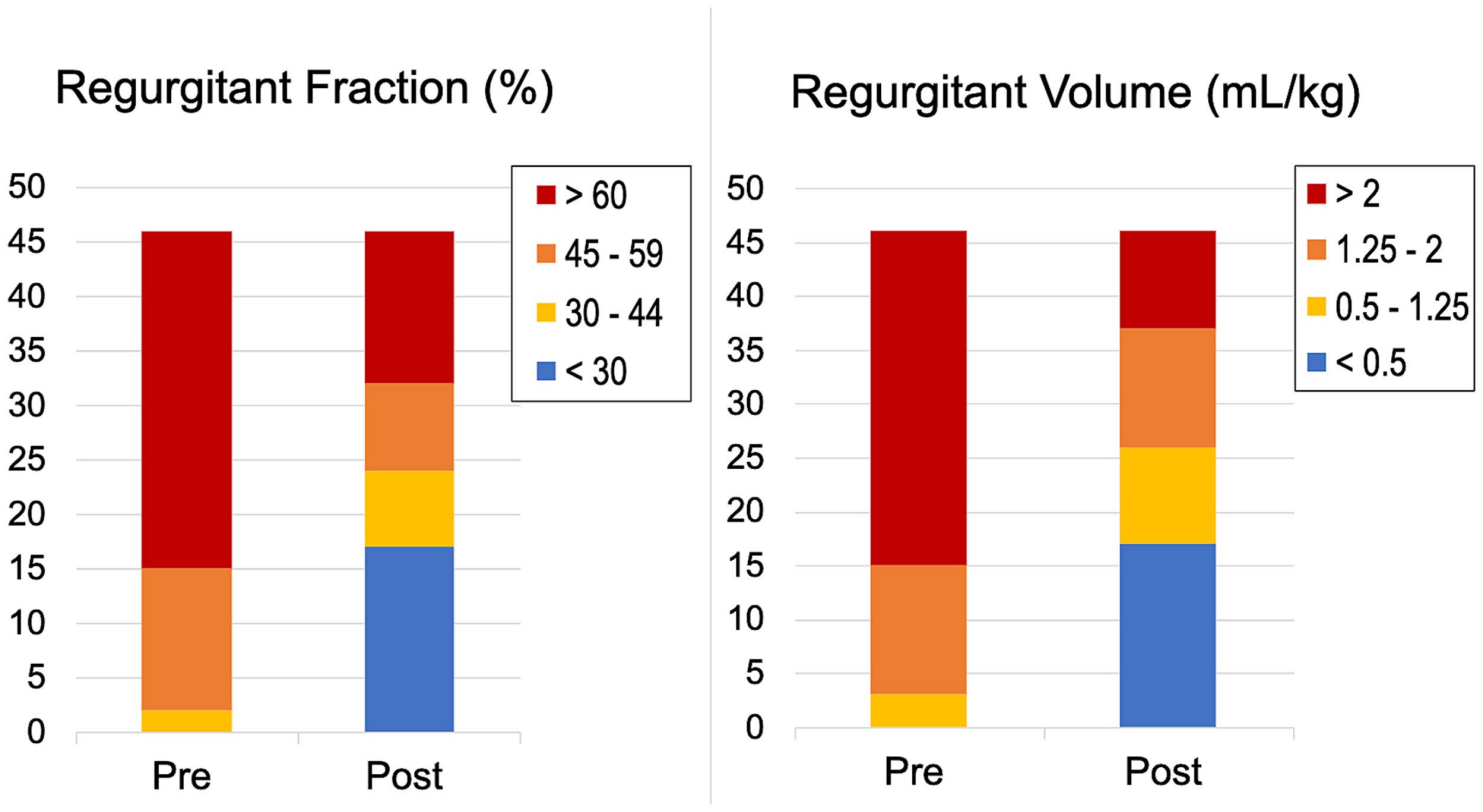
Distribution of regurgitant volume (mL/kg) and regurgitant fraction (%) severity before and at hospital discharge after transcatheter edge-to-edge mitral valve repair.

**Figure 6:**
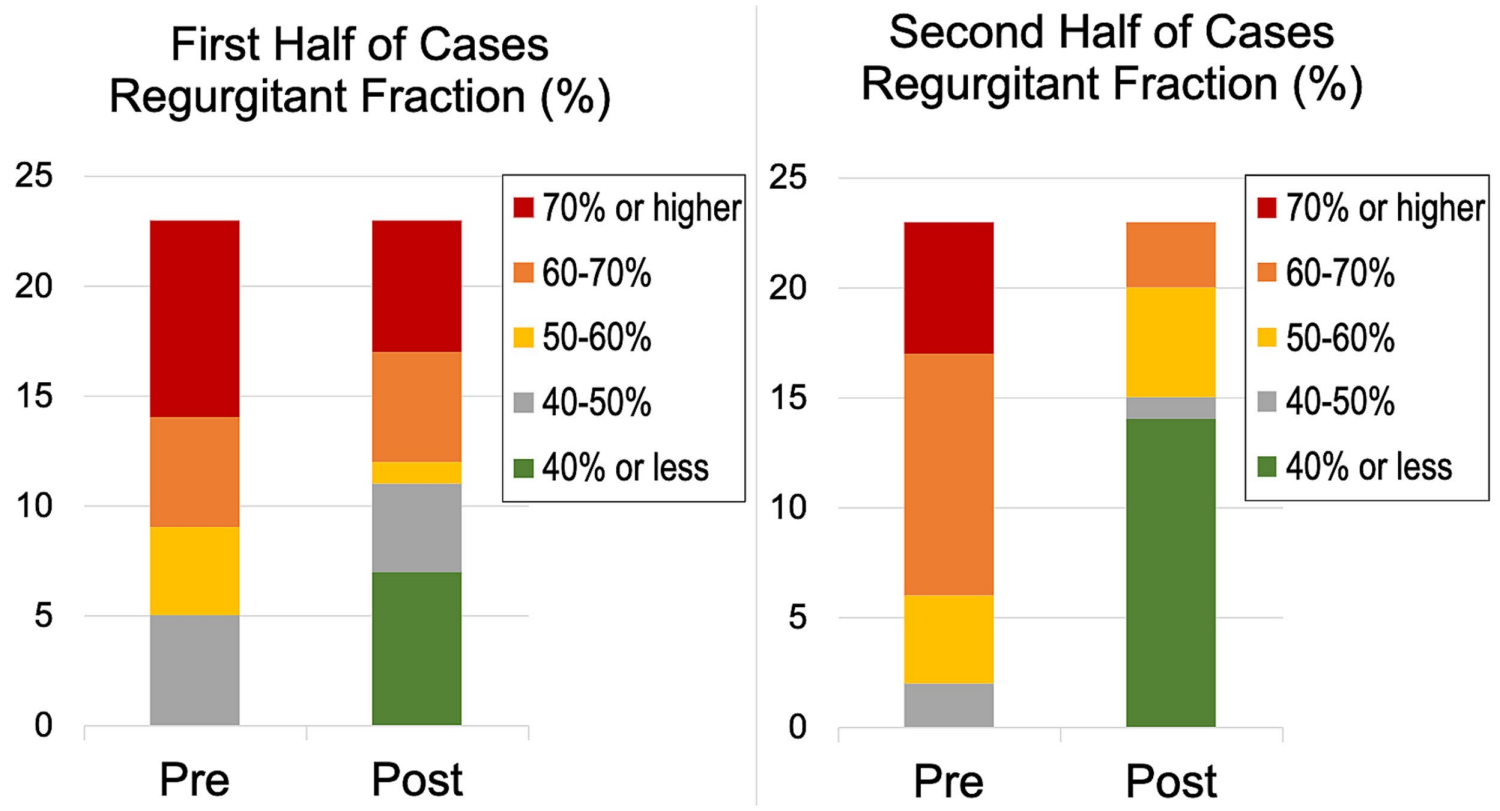
Distribution of regurgitant fraction (%) severity before and at hospital discharge after transcatheter edge-to-edge mitral valve repair for the first and second half of cases performed.

V-Clamps were deployed in the center of the valve at the A2 and P2 mitral segments resulting in a double orifice mitral valve (Figure 1B). Echocardiographic variables of cardiac remodeling and MR severity at baseline and hospital discharge for dogs undergoing deployment of at least one clamp and surviving to hospital discharge are reported in Table 2. Median percent reduction (%) [25th-centile, 75th-centile] in RVol and RF was 49.4% [87.7, 35.3] and 30% [78.2, −13.4], respectively. Distributions of RVol and RF severity before and after the procedure are shown in Figure 5. Median percent reduction in RVol in the second half of dogs in the study was 76.8% [93.0, −44.2] and was greater (*p* = 0.001) compared to the first half of dogs at 37.7% [52.7, −28.5]. Similarly, mean percent reduction (%) in RF in the second half of dogs in the study (53.2% ± 33.4) was greater (*p* = 0.001) compared to the first half of dogs (21.6% ± 28.7). Distributions of RF severity before and after the procedure for the first half compared to the second half of dogs in the study are shown in Figure 6. Intraprocedural hemodynamic and TEE spectral Doppler variables are reported in Table 3. Case example videos of TEE, fluoroscopy, and transthoracic echocardiography before and after TEER are shown (Supplementary Videos 5–7).

**Table 2:** Transthoracic echocardiographic variables before and after at discharge transcatheter edge-to-edge mitral valve repair (*n* = 46).

	Baseline	Post-procedure*	*p* value
Left atrial to aortic dimension ratio (short axis) (LA/Ao)	2.4 (2.0, 2.7)	2.2 (1.8, 2.6)	<0.001
Left atrial to aortic dimension ratio (long axis) (LA/Ao)	3.8 (3.4, 4.2)	3.5 (3.0, 4.0)	<0.001
LA volume end-systole (mL/kg)	4.8 (4.0, 5.6)	3.7 (2.7, 4.6)	<0.001
Left ventricular internal dimension in diastole (LVIDdN cm/kg^0.294^)	2.1 (2.0, 2.3)	2.0 (1.8, 2.1)	<0.001
LV end-diastolic volume (mL/kg)	4.6 (4.0, 5.5)	3.7 (2.8, 4.4)	<0.001
LV end-systolic volume (mL/kg)	1.0 (0.8, 1.5)	1.3 (0.9, 1.6)	0.004
Total stroke volume (mL/kg)	3.6 (3.2, 4.0)	2.2 (1.7, 3.0)	<0.001
Forward stroke volume (mL/kg)	1.3 (1.0, 1.5)	1.2 (1.0, 1.6)	0.35
Regurgitant volume (mL/kg)	2.3 (1.9, 3.1)	1.1 (0.3, 1.8)	<0.001
Regurgitant fraction (%)	65 (58, 76)	43 (14, 61)	<0.001
EROA (cm^2^/m^2^)	0.60 (0.40, 0.80)	0.25 (0.10, 0.50)	<0.001
Mitral inflow E-wave velocity (m/s)	1.2 (1.1, 1.4)	0.9 (0.8, 1.1)	<0.001
Mean mitral inflow pressure gradient (mmHg)	1.5 (1.0, 2.0)	1.5 (1.0, 2.3)	0.73
Heart rate (bpm)	120 (100, 132)	110 (90, 130)	0.07
Tricuspid regurgitation peak velocity (m/s)	2.7 (2.4, 3.2)	2.7 (2.4, 3.1)	0.08
Ejection fraction (%)	77.7 (71.6, 80.7)	63.5 (59.1, 69.6)	<0.001

Values reported as median (25th-centile, 75th-centile).

*Obtained 2 to 3 days post-procedure. EROA, effective regurgitant orifice area.

**Table 3:** Intraprocedural hemodynamic and spectral Doppler variables before and 5 min after transcatheter edge-to-edge mitral valve repair (*n* = 46).

	Before	After	*p* value
Heart rate (bpm)	89 (68, 98)	69 (60, 77)	<0.001
Blood pressure (Systolic)	110 (97, 122)	110 (99, 127)	0.27
Blood pressure (Diastolic)	58 (52, 62)	60 (53, 69)	0.03
Blood pressure (Mean)	74 (71, 79)	79 (71, 83)	0.03
Mean mitral inflow pressure gradient (mmHg)	0 (0, 1)	0 (0, 1)	0.31

Values reported as median (25th-centile, 75th-centile).

bpm, beats per minute.

All dogs were ambulatory within 24 h of the procedure. All dogs had ventricular ectopy post-procedure ranging from ventricular premature complexes to accelerated idioventricular rhythm in the first 48 h that was mostly self-limiting by the time of hospital discharge. Eight dogs were placed on sotalol therapy for ventricular arrhythmias prior to and at the time of hospital discharge. New-onset atrial fibrillation was documented post-procedure in four dogs. Two dogs spontaneously converted to a normal sinus rhythm within 24 h and two dogs converted to a normal sinus rhythm on sotalol therapy by the time of discharge. Two dogs experienced mild thrombosis of the valve/device within 24 to 48 h post-procedure. In one dog, the thrombus resolved within 24 h of initiation of oral apixaban (0.4 mg/kg q 8 h) and apixaban was discontinued after 48 h. In the second dog, the thrombus was reduced in size within 48 h after initiation of oral apixaban (0.4 mg/kg q 8 h) and was resolved one-month post-procedure. Median hospitalization for discharged dogs was 2 days.

## Discussion

Study design was based on U.S. Food and Drug Administration guidelines for traditional feasibility study of Class III medical devices (25). Primary endpoints were procedural feasibility and procedural safety. Short-term efficacy was a secondary endpoint. Early feasibility studies for medical devices are typically single-arm (uncontrolled) studies of target patients and typically represent the first experience with a new medical device and procedure. This study represented our entire initial experience with the second-generation V-Clamp device.

Procedural feasibility defined as the ability to deliver at least one clamp was 96%. Procedural failure in two dogs in this study was due to inability to simultaneously capture the anterior and posterior leaflets. Although both dogs were discharged from the hospital, one of these dogs had worsened MR and died suddenly shortly after discharge. The other dog was judged to be late-stage C and died of progressive heart failure a few weeks after the procedure. Operator inexperience and inappropriate case selection contributed to these failures. Anatomic factors that likely contributed to procedural failure were mid-systolic AP mitral valve annular diameter > 22 mm and bileaflet flail.

There were no procedural deaths. Two dogs were euthanized prior to hospital discharge due to worsened MR after the procedure. In one case, injury to the valve during the procedure could not be salvaged despite attempts to deploy three clamps. The other dog experienced avulsion of the head of the posteromedial papillary muscle attributed to injury by the introducer catheter which entered the heart caudal to the cardiac apex. Operator inexperience was judged to be a contributing factor in both these early adverse outcomes. One dog experienced detachment of the clamp from the posterior leaflet within 48 h attributed to failure to capture enough leaflet tissue within the clamp. Successful deployment of a second clamp 2 days after the initial procedure salvaged the situation, but this may not always be possible. An important strategy in avoiding leaflet detachment is measurement of the amount of leaflet capture by subtracting the residual leaflet lengths after grasping from the pre-procedural leaflet lengths with a goal of capturing ≥4 mm of leaflet tissue on each leaflet. The two instances of the clamp unlocking were attributed to operator inexperience and failure to adequately lock and test clamp locking. In the case that had an embolized clamp, retrieval of the clamp at the time of the procedure was not performed due to perceived challenges from the transapical approach. That patient remained clinically unaffected by the clamp and a second retrieval procedure was deemed unnecessary. Overall procedural success based on hospital discharge with at least one clamp was 92%. Procedural safety and overall success might be expected to improve with operator experience and improved understanding of appropriate case selection for edge-to-edge repair. Results of this study compare favorably with the EVEREST feasibility trial of MitraClip in humans which had an initial procedural failure rate of 20 to 25% (8, 9). In those studies, human patients could be rescued by open surgical valve repair or replacement which was not available for dogs in this study.

A welcomed outcome in dogs undergoing TEER was the low morbidity and quick recovery associated with this procedure. Dogs were able to be discharged from the hospital on the first or second post-operative day. Thoracostomy tubes were typically removed within 4 h of the procedure. Post-procedural bleeding was not observed. Most dogs experienced an accelerated idioventricular rhythm that was self-limiting without treatment. Transient atrial fibrillation was observed in four dogs that had converted back to sinus rhythm by the time of hospital discharge.

Overall procedural efficacy based on echocardiography before and days after the procedure varied based on disease stage, mitral anatomy and procedural experience. The average decrease in regurgitant volume was 54%. Procedural efficacy improved over the course of the study as reflected by the improved decreases in RVol and RF in the second half of the study compared to the first half. Procedural experience, earlier stage intervention, and an evolving understanding of appropriate case selection for edge-to-edge repair were likely factors in improved outcomes. All dogs in the study met the predetermined criteria for severe mitral regurgitation. Early cases in this study were at a more advanced ACVIM disease stage (late C and D) due to the investigational nature of this study. Stage B2 and early-stage C dogs were allowed to enter the study as operator confidence in the procedure was gained so long as they met criteria for severe MR.

Determining whether mitral valve anatomy is appropriate for edge-to-edge repair is a critical aspect of case selection in human patients undergoing TEER. Anatomic selection guidelines in humans have evolved based on experience in thousands of patients over a twenty-year period from simple binary guidelines in the EVEREST trial (9) to current classification schemes that stratify anatomic complexity as non-complex (ideal), complex (suitable), very-complex (challenging) and unfavorable (hard or impossible) (26). There were no predefined guidelines for anatomic complexity in this study other than predominate involvement of the central segments of the anterior (A2) and/or posterior leaflet (P2). Our understanding of case selection based on mitral valve anatomy evolved during this study, and this influenced case selection over the course of the study. Our subjective experience over the course of this study suggests that leaflet flail, particularity of the posterior leaflet, systolic A-P diameter > 20 mm, posterior leaflet length < 7 mm, shallow leaflet coaptation depth, and wide commissural vena contracta width all warrant caution in selecting dogs for TEER. These criteria will need to be further defined and validated over time.

Operator inexperience was likely a factor in outcomes over the course of this study as might be expected in a first feasibility study. Based on a large registry study of 275 TEER centers and 12,334 human patients, inflection points for procedural time, procedural success, and procedural complications occur at approximately 50 cases and continue to improve up to 200 cases (27). The results of this first study are encouraging given the inexperience of the operators. An optimistic view is that we might expect results to continue to improve as we gain procedural experience and better understanding of case selection. It is important to acknowledge that similar learning curves can be expected in dogs if this procedure is adopted more widely by new centers and operators.

Important questions regarding longer term outcomes remain unanswered by this initial clinical feasibility study. It remains to be determined to what degree reductions in MR severity will be sustained and result in improved survival and quality of life. Long-term follow up of human patients undergoing TEER in the pivotal EVEREST II demonstrated favorable survival and quality of life compared to patients undergoing surgical mitral valve repair, even though surgical repair resulted in better reductions in MR severity (10). These results have since been confirmed on meta-analysis of studies comparing TEER with surgical mitral repair (28). Long-term follow up of echocardiographic variables and survival of dogs in this study will provide further insight into the efficacy of TEER in dogs. Ultimately, a prospective pivotal study comparing medical therapy with and without TEER like the COAPT trial in humans (11) will be needed to fully understand the benefit of TEER in dogs.

This study has important limitations. The study was uncontrolled and only reports short-term efficacy. As such, it is not possible to draw conclusions about how TEER compares to established therapies for DMVD in dogs, including surgical mitral valve repair and medical therapy alone. Establishing long-term efficacy and benefit of TEER in dogs will require further study. This study did not have predefined selection criteria based mitral valve anatomy, but rather selection criteria based on mitral anatomy evolved over the course of the study and generally became more conservative. Continued experience will be needed to understand anatomic selection criteria for edge-to-edge mitral repair in dogs as was the case in humans undergoing TEER. Consensus guidelines for determining MR severity in dogs have not been established. As such, consensus guidelines and methods for determining MR severity in humans were used in this study which may or may not be appropriate for dogs.

In conclusion, initial feasibility results support continued development of TEER as a procedurally feasible, relatively low-risk, and low morbidity treatment for severe degenerative MR in dogs. Evidence of short-term efficacy is promising but will need verification with longer-term follow up. Operator experience, earlier intervention, and evolving understanding of favorable functional anatomy for edge-to-edge repair may result in the better reductions in MR severity in the future.

## Data Availability

The original contributions presented in the study are included in the article/Supplementary material, further inquiries can be directed to the corresponding author.

## Footnotes

^1^Canine Mitral Valve V-Clamp, Shanghai Hongyu Medical Technology Co., Shanghai, China.

^2^Philips EPIQ CVx, Philips Healthcare, Andover, Massachusetts.
