# Supplementary material for: Clinical feasibility study of transcatheter edge-to-edge mitral valve repair in dogs with the canine V-Clamp device
Source: Front Vet Sci. 2024 Dec 9;11:1448828. doi: 10.3389/fvets.2024.1448828 (PMC11663856; doi:10.3389/fvets.2024.1448828)
Supplement: Supplementary file 8 [file Data_Sheet_1.pdf]

frontiers

Supplementary Material

Supplemental Table A: Dog Breeds Included	
CKCS	14
Mixed Breed	14
Chihuahua	3
Dachshund	2
Shih Tzu	2
Havanese	2
Maltese	2
Beagle	2
Other Breeds: Bichon, Cocker Spaniel, Jack Russell Terrier, Japanese Chin, Miniature Poodle, Pomeranian, Scottish Terrier, Shetland Sheepdog, Tibetan Terrier	9
