# Supplementary material for: Clinical feasibility study of transcatheter edge-to-edge mitral valve repair in dogs with the canine V-Clamp device
Source: Front Vet Sci. 2024 Dec 9;11:1448828. doi: 10.3389/fvets.2024.1448828 (PMC11663856; doi:10.3389/fvets.2024.1448828)
Supplement: Supplementary file 9 [file Data_Sheet_2.pdf]

frontiers

Supplementary Material

Supplemental Table B: Procedural Failures, Deaths Before Hospital Discharge and Adverse Device-Related Events			
**Case #**	**Procedural Failures**	**ACVIM Stage**	**Follow-up**
2	Unable to grasp leaflets	C	Died congestive heart failure 44 days after discharge
34	Unable to grasp leaflets	C	Sudden death 48 hours after discharge
	**Deaths Before Hospital Discharge**		
15	Severe MR due to papillary evulsion	C	Euthanasia 3 days post-procedure
22	Worsened MR due to leaflet injury during procedure	C	Euthanasia 1 day post-procedure
	**Adverse Device-Related Events**		
5	Clamp opened after deployment but remained attached to leaflets. Second clamp successfully placed at time of index procedure.	B2	Alive 1133 days*
14	Single leaflet detachment 2 days post-procedure. Second clamp successfully placed 2 days after index procedure.	B2	Euthanized 637 days post procedure due to GI lymphoma
38	Clamp opened during procedure, detached, and embolized to ascending aorta. Second clamp successfully placed at time of index procedure.	B2	Alive 686 days*
**Days reported represent days alive until October 28th, 2024*			
