# Supplementary material for: Clinical feasibility study of transcatheter edge-to-edge mitral valve repair in dogs with the canine V-Clamp device
Source: Front Vet Sci. 2024 Dec 9;11:1448828. doi: 10.3389/fvets.2024.1448828 (PMC11663856; doi:10.3389/fvets.2024.1448828)
Supplement: Supplementary file 10 [file Data_Sheet_3.pdf]

Supplementary Material

VIDEOS:

1	Transapical Approach	Mini-thoracotomy is performed in the 7th intercostal space. Location of the transapical puncture site perpendicular to the mitral valve annulus is determined by blunt compression on biplane echocardiographic views. Two pledget-reinforced mattress sutures are placed and passed through tourniquets.
2	Single Clamp Transcatheter Edge-to-Edge Mitral Repair – Part 1	A 0.035" guidewire is passed into the left ventricle (LV) through a needle catheter. A 14Fr introducer is passed over the guidewire into the LV. The guidewire and dilator are exchanged for the mitral guide. The mitral guide is directed across the mitral valve into the left atrium (LA) under biplane echocardiographic guidance. The introducer is advanced into the LA.
3	Single Clamp Transcatheter Edge-to-Edge Mitral Repair – Part 2	Procedural steps for single clamp include passing the delivery device into the left atrium and orienting the clamp within the left atrium and bringing the lower arms of the clamp through the mitral valve.
4	Single Clamp Transcatheter Edge-to-Edge Mitral Repair – Part 3	Procedural steps for single clamp continued including capturing the leaflets by closing the upper arms of the clamp toward the mitral valve, confirming leaflet capture, and releasing the clamp.
5	Pre- and post-operative transesophageal echocardiography	Two- and three-dimensional *en face* transesophageal echocardiogram (TEE) images of the mitral valve in a 9-year-old Cavalier King Charles Spaniel pre- and post-transcatheter edge-to-edge mitral valve repair with the canine mitral V-Clamp device.
6	Fluoroscopic image post-transcatheter edge-to-edge repair of the mitral valve	Lateral fluoroscopic projection of the thorax post-transcatheter edge-to-edge mitral valve repair with the canine mitral V-Clamp device.
7	Pre- and post-operative transthoracic echocardiography	Two- and three-dimensional *en face* transthoracic echocardiogram (TTE) images of the mitral valve in a 9-year-old Cavalier King Charles Spaniel pre- and post-transcatheter edge-to-edge mitral valve repair with the canine mitral V-Clamp device.
